# Vaccine inequity and hesitancy: Dual factors in the emergence of novel SARS-CoV-2 variants

**DOI:** 10.1016/j.amsu.2021.103186

**Published:** 2021-12-23

**Authors:** Manish Dhawan, Priyanka, Ashima Sahni, Om Prakash Choudhary

**Affiliations:** Department of Microbiology, Punjab Agricultural University, Ludhiana, 141004, Punjab, India; The Trafford Group of Colleges, Manchester, WA14 5PQ, UK; Independent Researcher, 07, Type IV Quarter, College of Veterinary Sciences and Animal Husbandry, Central Agricultural University (I), Selesih, Aizawl, 796015, Mizoram, India; Department Political Science, Kanya Maha Vidyalaya, (Autonomous College), Jalandhar, 144004, Punjab, India; Department of Veterinary Anatomy and Histology, College of Veterinary Sciences and Animal Husbandry, Central Agricultural University (I), Selesih, Aizawl, 796015, Mizoram, India

**Keywords:** COVID-19, SARS-CoV-2, Variants, Emergence, Vaccine, Inequity, Hesitancy

Dear Editor

Since the commencement of the COVID-19 pandemic, several variants of concern (VOCs) such as Alpha (B.1.1.7), Beta (B.1.351), Gamma (P.1), and Delta (B.1.617.2) have been discovered and reported in various parts of the world. These viral mutants may have the potential to significantly affect the transmission of the virus, vaccine efficacy, therapeutic and diagnostic approaches. Furthermore, these mutations have led to the resurgence of consequent waves of the devastating COVID-19 pandemic throughout the world. In the midst of the major attempts to limit the COVID-19 pandemic, a highly mutated form of SARS-CoV-2 known as the Omicron variant (B.1.1.529) has been recently identified as a major threat [1]. According to the available data, the Delta and Omicron variants were discovered in India and South Africa, respectively, and both nations had poor vaccination rates at the time of variant emergence. Furthermore, the questionable efficiency of the healthcare system and the prevalence of vaccine hesitancy in developing countries may have a significant role in the emergence of variants.

Several experts have speculated that the new SARS-CoV-2 variants may evolve at different times. Looking deeper into the causes of the emergence of the SARS-CoV-2 variants, the vaccine hesitancy and inequities in the vaccine distribution amongst the developing *vs.* developed nations might be associated with the emergence of the SARS-CoV-2 variants. Immunocompromised people, with their weakened immune systems and increased vulnerability to infection as a result of a lack of proper public health infrastructure, lower vaccination rates, and other variables, have recently been hypothesized to accelerate the emergence and spread of novel variants of SARS-CoV-2, which may worsen the situation [2].

Vaccine hesitancy is one of the most major threats to the global health since it jeopardizes our potential to eliminate infectious diseases *via* the generation of herd immunity among the whole population through vaccination. Individuals who refuse to be vaccinated or opt-out of COVID-19 vaccination may slow the overall vaccination pace as well as coverage, resulting in lower vaccination rates, and obstructing the global efforts to control the spread of SARS-CoV-2, as unvaccinated individuals can act as SARS-CoV-2 reservoirs, causing more outbreaks [3].

Furthermore, the disparities in the vaccine distribution amongst the countries have been recognized as another challenge, leading to the evolution of SARS-CoV-2 variants [4]. As an example, only around a fifth of Africans, including millions of healthcare professionals and vulnerable communities, are completely immunized against the COVID-19. There are still numerous unprotected susceptible communities across the world that require vaccination on a priority basis. It has been postulated that in the future other VOCs might emerge if the SARS-COV-2 continues to circulate especially in the unvaccinated masses, and the resulting viral evolution may eventually lead to vaccine-resistant variants.

Low vaccination rates in certain parts of the world, notably the developing countries, including Africa, are one of the important causes contributing to the emergence of VOCs. Therefore, there is an urgent need to acquire enough COVID-19 vaccines and ensure a regular supply to the countries with significantly lower vaccination rates [4]. Furthermore, it has been suggested that a slow pace of vaccination may result in an increase in COVID-19 cases and associated deaths, as well as the possibility of a longer epidemic. This necessitaes the need for a high rate of vaccine uptake which can be achieved through increased vaccine production, as well as rapid and efficient transport, storage, and distribution monitoring. Substantial herd immunity to SARS-CoV-2 can only be achieved by vaccination rather than through natural infection alone. Hence, worldwide efforts should be concentrated on creating a vaccination programme using a highly effective vaccine with the goal of achieving the highest feasible coverage [5].

Finally, it can be concluded that even after the development of efficient vaccines against COVID-19, there are still major challenges, such as the prevalence of vaccine hesitancy and inequities in the distribution of COVID-19 vaccines. These challenges should be seriously considered and addressed by all countries to halt the circulation of SARS-CoV-2 among the worldwide population and stop the emergence of novel SARS-CoV-2 variants.

## Sources of funding

This study received no specific grant from any funding agency in the public, commercial, or not-for-profit sectors.

## Ethical approval

This article does not require any human/animal subjects to acquire such approval.

## Consent

Not applicable.

## Author contribution

**Manish Dhawan:** Conceptualization, Data Curation, Visualization, Writing - Original Draft, Writing - review & editing. **Priyanka:** Writing - Original Draft, Writing - review & editing. **Ashima Sahni:** Writing - review & editing. **Om Prakash Choudhary:** Supervision, Writing - Original Draft, Writing - review & editing.

## Registration of research studies

1. Name of the registry: Not applicable
2. Unique Identifying number or registration ID: Not applicable
3. Hyperlink to your specific registration (must be publicly accessible and will be checked): Not applicable

## Guarantor

Om Prakash Choudhary, Assistant Professor, Department of Veterinary Anatomy and Histology, College of Veterinary Sciences and Animal Husbandry, Central Agricultural University (I), Selesih, Aizawl-796015, Mizoram, India. Tel: +91-9928099090; Email: dr.om.choudhary@gmail.com.

## Declaration of competing interest

All authors report no conflicts of interest relevant to this article.
